# Metabolic and Respiratory Costs of Increasing Song Amplitude in Zebra Finches

**DOI:** 10.1371/journal.pone.0023198

**Published:** 2011-09-07

**Authors:** Sue Anne Zollinger, Franz Goller, Henrik Brumm

**Affiliations:** 1 Communication and Social Behaviour Group, Max Planck Institute for Ornithology, Seewiesen, Germany; 2 Department of Biology, University of Utah, Salt Lake City, Utah, United States of America; 3 School of Biology, University of St Andrews, St Andrews, Fife, United Kingdom; University of Plymouth, United Kingdom

## Abstract

Bird song is a widely used model in the study of animal communication and sexual selection, and several song features have been shown to reflect the quality of the singer. Recent studies have demonstrated that song amplitude may be an honest signal of current condition in males and that females prefer high amplitude songs. In addition, birds raise the amplitude of their songs to communicate in noisy environments. Although it is generally assumed that louder song should be more costly to produce, there has been little empirical evidence to support this assumption. We tested the assumption by measuring oxygen consumption and respiratory patterns in adult male zebra finches (*Taeniopygia guttata*) singing at different amplitudes in different background noise conditions. As background noise levels increased, birds significantly increased the sound pressure level of their songs. We found that louder songs required significantly greater subsyringeal air sac pressure than quieter songs. Though increased pressure is probably achieved by increasing respiratory muscle activity, these increases did not correlate with measurable increases in oxygen consumption. In addition, we found that oxygen consumption increased in higher background noise, independent of singing behaviour. This observation supports previous research in mammals showing that high levels of environmental noise can induce physiological stress responses. While our study did not find that increasing vocal amplitude increased metabolic costs, further research is needed to determine whether there are other non-metabolic costs of singing louder or costs associated with chronic noise exposure.

## Introduction

Songbirds, like most organisms that use acoustic signals, are faced with the need to communicate in an increasingly noisy world. As anthropogenic noise levels rise, the question of what, if any, are the physiological costs of living and communicating in noise becomes an increasingly critical one. High levels of anthropogenic noise have deleterious effects on health and development in mammals [1], [2], [3] and impact on the breeding success [4], vocal behaviour [5] and community structure of birds [6], [7]. One of the best-documented impacts of environmental noise is a change in vocal behaviour of songbirds and other taxa that rely on acoustic signals to communicate. There are several strategies animals may use to increase signal efficacy over background noise, including changing the temporal pattern of their vocalizations, shifting the frequency of the signal to minimize masking by noise, and increasing vocal amplitude to improve the signal-to-noise ratio [8].

In addition to ecological demands for signal transmission, song amplitude may also be an honest signal of current condition in songbirds. Bird song is an important signal in mate attraction and in many species, including zebra finches, song is a key trait used by females to select a mate [9], [10]. Several aspects of song quality have been shown to be indicators of past or present condition of the male, suggesting that song may be used as an honest signal of male quality. In some species, song types thought to be the most challenging to produce are those deemed most attractive by females in mate choice experiments. In addition to various other measures of vocal complexity and performance, song amplitude has recently been found to also influence female preferences in zebra finches (*Taeniopygia guttata*), and may reflect current condition in males. Ritschard et al. [11] found that female zebra finches prefer higher amplitude song, and chose songs that were louder at source even if they were the same amplitude at the position of the female. In another study, Ritschard & Brumm [12] manipulated the body condition of adult male zebra finches and found that song amplitude decreased as body weight, a measure of current condition, decreased.

So, if a relatively small rise in amplitude both increases the communication distance and make songs more attractive, shouldn't birds sing at peak amplitude all the time? While there are few studies that actually measure the range of song amplitudes that individual birds can produce, especially in the field, there is evidence from a variety of species that suggests most birds do not sing at the extremes of their vocal abilities and can increase vocal amplitude in response to changes in social or environmental conditions. The paucity of such data is due in large part to the technical difficulty of accurately measuring source amplitude in the field, since the distance to and orientation of the sound source both significantly affect the accuracy of the measurements. For example, male nightingales (*Luscinia megarhynchos*) sing more quietly when singing alone than when singing in male-male interactions [13], and zebra finches increase their signal amplitude as the distance to the receiver increases [14]. In addition, several species of birds, including nightingales and zebra finches, have been shown to increase vocal amplitude in response to increases in background noise levels [15], [16], a phenomenon known as the Lombard effect (reviewed in [8]). In several species of songbirds, individuals living in areas with high levels of anthropogenic noise have been found to sing with higher minimum frequencies than those living in quieter areas (e.g. [17], [18], [19], [20]). Although song amplitude was not measured in these studies, an increase in pitch is a common component of the Lombard effect (e.g., [21], [22]), and so these studies may be describing a by-product of singing louder in response to noise. However, some authors have hypothesized that these frequency shifts may be a less costly way to increase communication distance than singing more loudly. That song amplitude may be a signal of current condition suggests that the production of louder songs might be more costly to produce than quieter songs. Although increasing levels of anthropogenic noise or selective pressure from a female preference for louder songs may push males to sing louder, little is known about the costs or constraints on vocal amplitude in birds. In particular, the metabolic and respiratory control of vocal amplitude adjustment has not previously been investigated.

Song in birds has been shown to be relatively cheap in terms of metabolic energy consumption, especially when compared with the energy required for other common activities such as flight [23], [24], [25], [26], [27], [28], however, the metabolic costs of song under natural conditions may be higher than that measured in the laboratory [29]. One reason for this is that most previous measurements of the metabolic cost of song have been on birds singing at “normal” or “comfortable” vocal amplitudes, or at least in situations with low levels of ambient room noise. Moreover, songbirds in sound shielded rooms, as commonly used in bioacoustic experiments, may sing at lower song amplitudes than in the wild [30]. Like bird song, human speech at normal, comfortable sound pressure levels is not an energetically expensive activity, but a study of metabolic costs of amplitude adjustments in human vocalization found that louder speech was considerably more costly [31]. When instructed to vocalize at a “comfortable” sound pressure level (SPL) in a quiet room, humans only required energy levels similar to that of sitting and reading silently. Even when speaking continuously for 7 minutes at normal amplitudes, ventilatory homeostasis and blood acid-base balance was maintained. However, when asked to repeat the same vocalizations at higher SPL levels (+10 dB above comfortable levels) energy expenditure significantly increased and homeostasis was disrupted [31]. Oberweger and Goller [25] likewise observed that quiet song, in a starling (*Sturnus vulgaris*) singing in a small respirometry chamber, required lower rates of oxygen consumption than loud song. However, in this bird the song structure was markedly different between the two conditions, the quiet song being composed of “loose sequences of soft whistles”, compared to the loud song of “snarls, hisses and whistles.” So it is not clear if the change in energy requirements was related to differences in song structure or song amplitude. In addition, as soft song was quieter than the “normal” song amplitudes of captive starlings, the observation suggests more that soft song may require less energy than normal amplitude songs, rather than that loud song required more. Whether singing the same song types at higher amplitude significantly increases metabolic energy expenditure in songbirds is not known.

In addition to a potential increase in metabolic energy required for louder song, there are other possible physiological costs of increasing vocal amplitude. In human voice production, the primary mechanism used to increase vocal amplitude is to increase lung pressure, thereby increasing aerodynamic power at the larynx. In addition, two other mechanisms are used in humans to increase amplitude without necessarily increasing lung pressure. These are (a) adjusting the width of the glottis to maximize the amount of aerodynamic power that is converted into acoustic power and (b) adjusting the vocal tract to match the frequency of the source and so boost sound energy at that frequency. However, none of these three mechanisms are entirely independent of sound frequency; and changes in vocal amplitude are often coupled with changes in pitch [32], [33]. While it is likely that songbirds can use similar mechanisms to adjust song amplitude the experimental evidence for amplitude regulation in songbirds is less direct than it is in humans. In zebra finches with experimentally reduced air-sac volume, both the air sac pressure, rate of air flow and sound amplitude were found to decrease [34]. In another study, relative levels of sound amplitude within syllables were found to be positively correlated with higher air sac pressure and larger beak gape in zebra finches although the absolute sound pressure levels and corresponding air sac pressure values were not measured [35]. These studies suggest that air pressure and rates of air flow through the syrinx regulate vocal amplitude in birds in much the same way as they do in humans, but the role of subsyringeal pressure on regulating vocal amplitude has not been investigated in detail. If songbirds do increase the rate of air flow and/or increase air sac pressure in order to increase vocal amplitude, louder song will require higher volumes of air from the lungs than quieter song. Birds singing at high amplitudes would then presumably exhaust their lung capacity sooner during loud songs than during quieter songs. There are several ways in which a songbird might compensate for increased air expenditure, including taking deeper or longer minibreaths (which might translate into longer between-syllable, or between-motif intervals), or reducing song rate.

Since increasing song amplitude is generally assumed to come at a cost [36], and may be a sexually-selected signal of male condition, we investigated how energy expenditure and respiratory patterns vary with song amplitude. In particular, we studied how increasing song amplitude affects oxygen consumption, air sac pressure minibreath behaviour and song bout duration in a small songbird, the zebra finch.

## Results

We recorded oxygen consumption using the small helmet respirometry mask from seven adult male zebra finches, however only three birds sang in the helmet at more than one vocal amplitude and at different background noise conditions. From these 3 remaining birds we compared rates of oxygen consumption (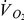) during song, low-level activity and inactivity at a range of amplitudes and in multiple background noise conditions. Background noise conditions at which all three birds sang included no noise playback (ambient room noise, including the noise of the air pump), measured at 54 dB(A) inside the helmet; intermediate noise (74 dB(A)) and high noise (80 dB(A)). In addition, two of the birds sang at a fourth background noise level (66 or 68 dB(A)). Data on song amplitude with bout duration and on air sac pressure with minibreath size were recorded from an additional six and five un-helmeted birds, respectively (Table 1).

**Table 1:** Birds used in the various experiments reported herein.

ID	year	Breeding stock	Body mass (g)	Helmet	Uncalibrated Air Sac Pressure	Calibrated Pressure vs. amplitude	Lombard effect - helmet	Lombard effect - no helmet	Song duration vs. amplitude	Minibreath analysis	Post-song apnea
**BFP**	2008	US	12.7					+			
**G16**	2008	US	14.0	+	+		+		+		
**G18**	2008	US	13.1	−	−		−	+	+		
**G33**	2008	US	13.7					+			
**G36**	2008	US	12.8						+		
**N36**	2008	US	13.9					+			
**Z12**	2008	US	14.5					+			
**B86**	2010	US	13.9			+		−		+	+
**G62**	2010	UK	18.2	+		+	+		+	+	+
**G65**	2010	UK	17.2	−		+	−	−		+	−
**G66**	2010	UK	18.5	−		−		−			−
**G68**	2010	UK	18.6	+		+	+		+		+
**G79**	2010	UK	17.9			+		−			−
**RW1**	2010	US	14.2			−		−		+	−
**V13**	2010	US	13.1			−				+	+
**Y42**	2010	US	13.5	−		−		+	+	+	−

Plus signs (+) indicate data from this bird for this treatment were used in analysis. Minus signs (−) indicate that birds were subject to the experimental treatment, but sang only at a single background noise level, or yielded otherwise unusable data and were not included in analyses. An additional four birds in 2008 and three birds in 2010 were used in either helmet or air sac pressure experiments, but did not sing in any condition and are not included here.

### Noise-related changes in song amplitude and bout duration

All three helmeted birds significantly increased the sound pressure level of their songs in response to each increase in background noise levels (Kruskal-Wallis One Way ANOVA on Ranks, bird G16: H = 163.55, df = 3, P<0.001; bird G62: H = 15.41, df = 2, P<0.001; bird G68: H = 49.37, df = 3, P<0.001, with all pair-wise comparisons between intermediate steps in background noise levels within birds, made using Dunn's Method, significant at P = 0.05 or lower). Song bout duration was not significantly correlated with the SPL of song or with background noise levels in either helmeted or un-helmeted birds (Table 2).

**Table 2:** Duration of song bouts and peak song amplitudes for each background noise condition, and correlation coefficients of song amplitude and bout duration for each individual.

			Background noise level					
Bird ID	N	Min – Max	50–54 dB	66–68 dB	70–74 dB	80–86 dB	*ρ*	P
G16*	57	Song amplitude (dB(A))	73.6–82.5	92.2–94.1	97.0–107.6	108.3–115.1	0.057	0.675
		duration (s)	3.07–13.49	1.95–5.16	2.88–14.38	3.63–13.51		
G62*	13	Song amplitude (dB(A))	86.5–86.4	-	94.9–96.1	106.2–107.7	0.183	0.550
		duration (s)	1.63–1.65	-	1.02–2.94	0.81–2.08		
G68*	55	Song amplitude (dB(A))	80.1–81.4	92.5–94.8	96.0–97.1	102.2–109.1	0.048	0.728
		duration (s)	0.65–3.97	0.69–5.48	0.98–4.67	0.84–2.99		
G18	21	Song amplitude (dB(A))	76.1–88.4	90.8–95.6	96.9–98.0	100.1–100.2	0.047	0.840
		duration (s)	1.49–5.35	0.51–6.96	1.76–7.16	2.69–3.47		
G36	60	Song amplitude (dB(A))	75.1–81.7	91.2–94.3	87.5–99.4	101.9–103.9	0.110	0.401
		duration (s)	0.78/7.28	1.05–6.67	0.29–2.75	1.06–2.28		
Y42	33	Song amplitude (dB(A))	89.0–89.9	-	90.1–96.1	98.1–107.0	0.251	0.128
		duration (s)	0.56–2.46	-	1.97–4.35	0.36–7.19		

* Recordings made while bird was in the respirometry helmet.

**Figure 1:**
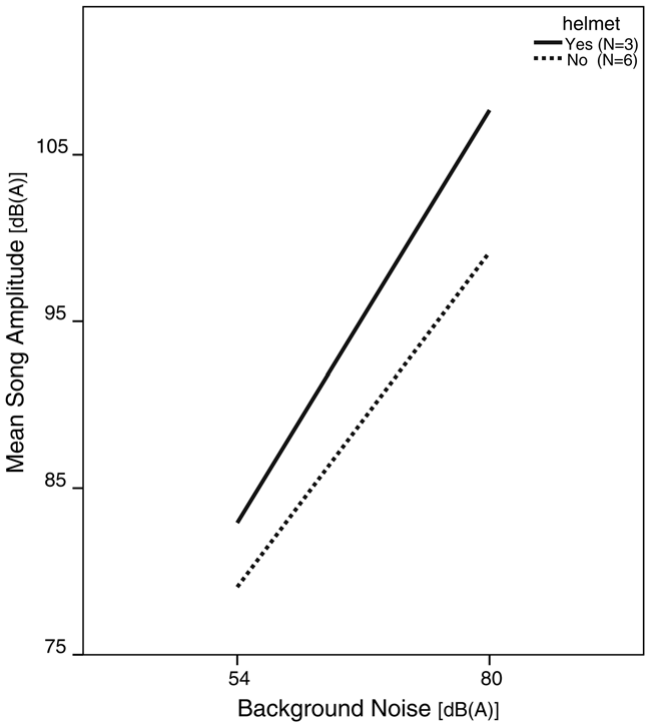
Mean increase in song amplitude between the lowest (ambient room noise) and highest background noise conditions. Solid line is the change in mean song amplitude for three birds recorded inside respirometry helmets. Dashed line is the change in mean amplitude for six unhelmeted birds recorded in small plastic chambers with the same microphones as those used in respirometry helmets. For each individual, the microphone was positioned in a fixed location relative to the bird's head. In both the chambers and the helmets, the microphones were positioned very close (less than 2.5 cm) to the source, but the microphones inside the helmets were typically nearer to the bird's head than were the chamber microphones.

Wearing the helmet did not inhibit the Lombard effect (Figure 1), inasmuch as the increase in song amplitude was the same, or may even be greater than that observed in zebra finches recorded without helmets at the same background noise conditions (GLMM; Mean Sq. = 21.88; df = 1; F = 4.82, P = 0.06). The recorded song amplitudes were higher on average in the helmet-wearing birds, but this difference is most likely due to the closer proximity of the microphone to the birds heads compared to the microphone placement in the non-helmet sound recording set-up. We therefore only compared the slope of the change in amplitude between the low and high noise conditions rather than comparing the absolute amplitude between groups. Considering our small sample size, it is certainly possible that despite the marginally non-significant p-value, helmeted birds do show an even stronger Lombard effect than unhelmeted birds. However, although the mean difference between the low and high song amplitude in helmeted birds (24.8 dB) was greater than in unhelmeted birds (20.1 dB) there was considerable overlap in the 95% confidence intervals of these mean differences (15.35–34.16 for helmeted birds, and 17.30–22.85 for unhelmeted birds). So the apparent difference in slopes, or degree of change in response to noise should be viewed with caution. In addition, although efforts were made to recreate a similar recording environment for the unhelmeted birds (see Methods), differences in slopes of increased amplitude may actually reflect differences in recording conditions, such as sound reflection from the inside of the helmet or acoustic effects resulting from the closer proximity of the microphone, rather than a biological difference in song amplitude per se. Finally, the primary goal of this test was to confirm that the birds in helmet did sing louder in response to noise. So in the end, it is less important if the degree of change may be slightly different from that of unhelmeted birds than the demonstration that helmeted birds did indeed respond to background noise with significant increases in song amplitude.

### Air sac pressure and respiratory adjustments for loud song

**Figure 2:**
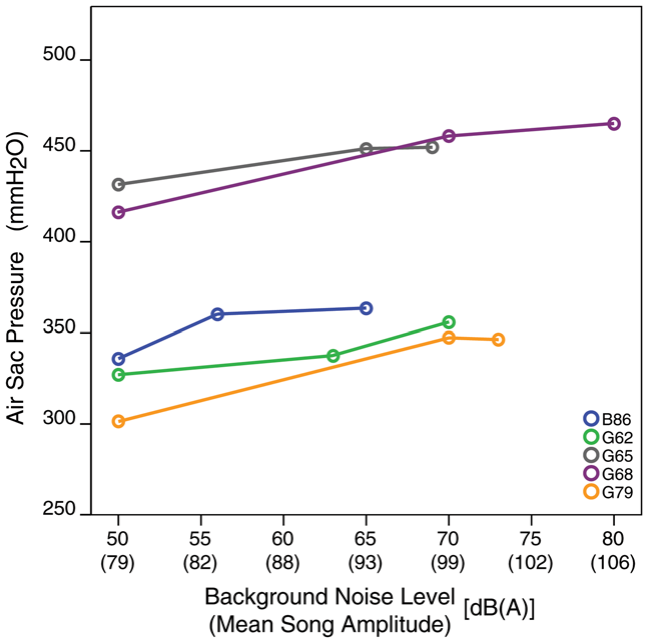
Subsyringeal air sac pressure increased with increased song amplitude. The subsyringeal pressure required for loud song (highest background noise condition) was significantly greater than pressure required to produce quieter song (no background noise).

Calibrated air sac pressure measurements were recorded from five birds singing at different vocal amplitudes. In all five birds subsyringeal pressure was significantly greater during songs produced in the highest background noise condition than during production of the same syllables at low SPL levels (exact Wilcoxon signed ranks test: T = 0, N = 5 birds, P = 0.03) (Figure 2). Pressure differences between quiet and intermediate, or between intermediate and loud songs were not always statistically significant for individual birds as was the difference between the quietest and loudest songs.

The duration and depth of minibreaths ( by integrating the area under the curve of the pressure trace as it dropped below atmospheric pressure between successive song syllables during song bouts) did not differ significantly between songs of different amplitude in five of six birds. Even between the lowest and highest song amplitudes, we did not find differences in minibreath duration (*bird B86*: U = 392, T_(20, 42)_ = 657, P = 0.68; *bird G65*: U = 549, T_(12, 92)_ = 633, P = 0.98; *bird G62*: U = 354, T_(15, 39)_ = 351, P = 0.30; *bird RW1*: U = 636, T_(28, 59)_ = 1422, P = 0.085; *bird Y31*: U = 599, T_(20, 50)_ = 611, P = 0.20) for five of the six birds. However one bird did have significantly longer minibreaths during louder songs than during quieter songs (*bird V13*: median durations low = 0.0361 s, high = 0.0372 s, U = 631, T_(30, 30)_ = 734, P = 0.008. Minibreaths were also slightly, but significantly, deeper during high amplitude songs than during low amplitude songs in one bird (*bird G62*: U = 400, T_(15, 39)_ = 305, P = 0.04) but did not differ in the remaining 5 birds (*bird B86*: U = 412, T_(20, 42)_ = 638, P = 0.91; *bird G65*: U = 560, T_(12, 92)_ = 622, P = 0.94; *bird RW1*: U = 401, T_(17, 26)_ = 585, P = 0.09; *bird V13*: U = 106, T_(14, 22)_ = 195, P = 0.730; *bird Y31*: U = 412, T_(20, 50)_ = 798, P = 0.26 For bird RW1, the median difference in both minibreath duration and minibreath depth were approaching significance statistically. Given the limited sample size, which was limited by the short time during which the air sac pressure recordings were reliable post-implantation, the non-significant results should be interpreted with caution. Some birds (four of nine we examined, Table 1) regularly exhibited brief periods of apnea immediately after singing (as described in Franz and Goller, 2003). However, in these four birds there was no significant effect of song amplitude (χ^2^ = 1.16 df = 1, P = 0.28), or song duration (χ^2^ = 0.56, df = 1, P = 0.45) on the duration of post-song apnea, nor did we find a significant interaction between song amplitude and song duration on the duration of the apnea events (χ^2^ = 0.16, df = 1, P = 0.69).

### Oxygen consumption during song at different amplitudes

**Figure 3:**
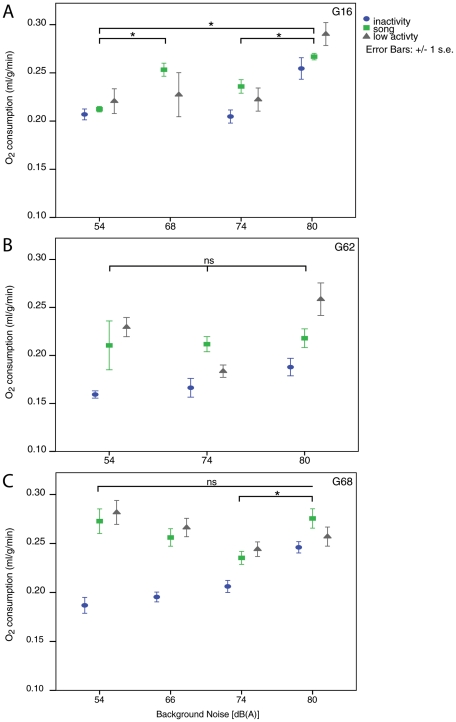
Rates of oxygen consumption during three categories of behaviour: song (green squares), low-level activity (grey triangles) and inactivity (blue circles) at different background noise conditions for three individuals. A) Rates of oxygen consumption were significantly higher during the quietest and loudest songs in only one bird, G16. However, the increase in oxygen consumption during periods of inactivity and low-level activity at the same background noise conditions were just as great, suggesting that the increase may not be due simply to the increase in song amplitude. B and C) Rates of oxygen consumption at low, intermediate and high background noise levels and song amplitudes were not significantly different in birds G62 or G68. All three birds showed an increase in oxygen consumption during periods of inactivity as background noise increased. Asterisks denote significance at p<0.01 levels for mean differences between means for rates of oxygen consumption during song at different amplitudes.

While sound amplitude (SPL) and subsyringeal air sac pressure during song increased significantly in response to each increase in background noise, oxygen consumption during song did not follow the same clear pattern. In two birds, G62 and G68, 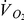 during the loud songs (mean song amplitude = 108.4 and 108.5 dB(A), respectively) did not differ significantly from 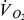 during normal amplitude songs (mean amplitude 88.0 and 80.8 dB(A)) despite differences in SPL of more than 20 dB (Figure 3b & 3c). Bird G16 had a significantly higher 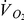 during loud song than during normal amplitude song (Mann-Whitney U test: U = 113, N_hi_ = 26, N_no_ = 91, P<0.001). Song at two intermediate amplitude levels was measured for bird G16, and 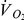 between these two conditions (background noise 68 and 74 dB(A), mean song amplitudes 93.5 and 97.8 dB, respectively) did not differ, but both differed significantly from 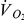 during normal and loud song (Figure 3a).

Oxygen consumption during intermediate-amplitude songs did not differ from normal or loud song for bird G62. Bird G68 had a significantly lower 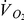 during intermediate song (background noise 74 dB(A), mean song amplitude 99.04 dB(A)) than during either loud or normal amplitude song (Mann-Whitney U-Test: 54–74 dB comparison: U = 126, N_54_ = 16, N_74_ = 29, P = 0.012; 74–80 dB comparison U = 52, N_74_ = 29, N_80_ = 9, P = 0.007), although songs produced during the two intermediate background noise conditions for this bird (66 and 74 dB) did not differ significantly in energy requirements from each other (U = 144, N_66_ = 15, N_74_ = 29, P = 0.07). We were only able to obtain recordings of this bird singing at intermediate levels during the last hour of a 4-hour recording session, after noise levels had been reduced from 80 to 74 dB, so the drop may be due to an order effect, as the bird had several hours in the helmet to habituate both to the helmet and the louder background noise.

**Figure 4:**
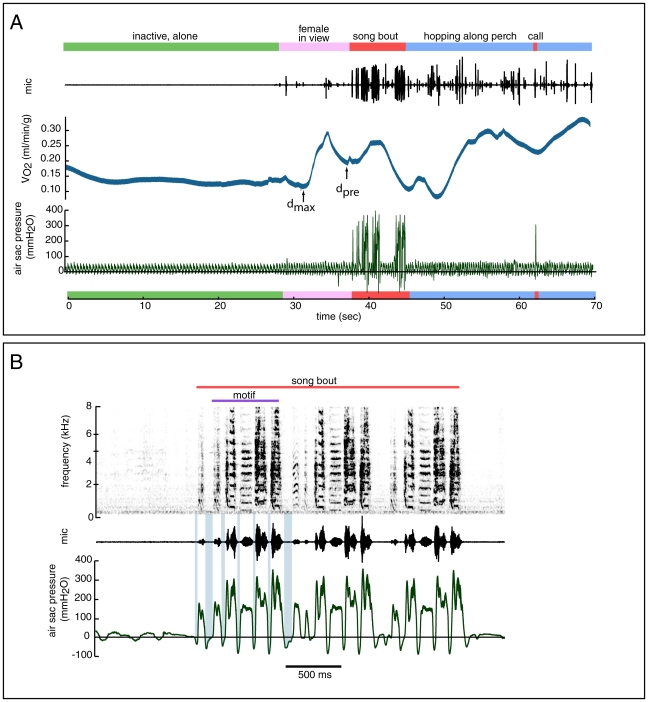
Rates of oxygen consumption (blue trace, panel A) varied with activity and with background noise conditions. In this typical example (A) the bird is sitting quietly out of view of any conspecifics during the first 30 seconds. Both 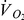 and the respiration rate remain fairly constant (each cycle from positive to negative in the air sac pressure trace (bottom, green) represents an expiration and inspiration during quiet respiration). Around 30 sec, a female is moved into the visual range of the subject bird. With the presence of the female, respiration rate increases and 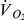 increases after a short lag. Immediately before song, there is a drop in 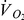. Measurements of pre-Song to during-Song differences were made from the minimum point that occurred within 500 ms of the onset of song (d_pre_), and also from the lowest 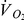 rate observed in the 10 second interval before the onset of song (d_max_), to the peak 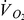 during the song bout. (B) An example of the air sac pressure pattern (bottom trace, green) during a typical zebra finch song bout (spectrogram, top), in this song the motif is repeated 3times. Minibreaths (seen as periods of negative pressure between syllables, and shaded blue in the first motif for illustration purposes) did not change in depth or duration with increases in song amplitude in 4 out of 5 birds measured.

**Figure 5:**
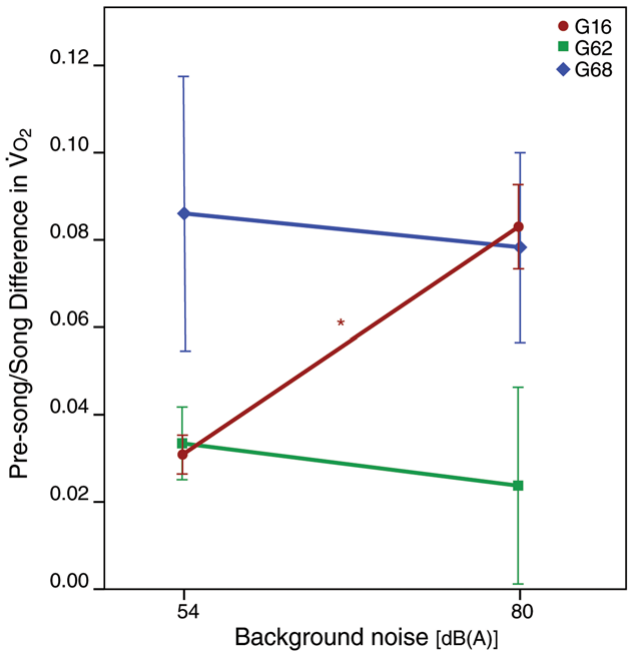
Differences between the mean rate of O_2_ consumption during song and the mean rate of O_2_ consumption (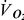) immediately before song bouts. In one bird (G16), song amplitude and background noise condition were significantly correlated with differences between pre-song and during-song 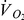 levels. The remaining two birds (G62 and G68) pre-song to during-song differences were not correlated with background noise treatment or song amplitude. Data points show mean difference between pre-song and during-song 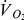 levels, error bars indicate 95% confidence intervals (* indicates significance at p<0.001 levels, see text for details).

In most cases, song bouts were immediately preceded by a sharp decrease in 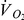 (Figure 4), although activity and 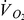 levels often varied greatly in the 10 s period prior to singing. We compared the difference between 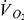 levels during song and 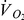 levels during the 500 ms interval pre-song, for low and high amplitude songs (at 54 and 80 dB background noise conditions) (Figure 5). In one bird (G16) we found a significant linear correlation between song amplitude and the difference between pre-song and during-song 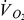 levels (R = 0.70, F_(1, 115)_ = 107.3, P<0.001), but in the other two birds (G62 and G68) there was no significant relationship between song amplitude and the difference between pre-song and during-song 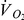 values (G62: R = 0.25, F_(1, 8)_ = 0.46, P = 0.52; G68: R = 0.06, F_(1, 23)_ = 0.07, P = 0.79).

We also compared differences between 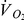 during song and during the 500 ms period with the lowest 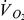 level that occurred during the 10 seconds prior to the onset of song (max 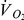 difference, Figure 4), at high and low song amplitudes (50 and 80 dB background noise conditions). The “max 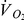 difference” at 50 dB and 80 dB conditions followed a similar pattern in the 3 birds to that observed in the change in pre-song 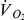 and during-song 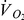 differences.

### Song vs. inactivity

Overall, 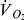 during song was greater than 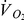 during inactivity for all three birds (G16: U = 1478, N_so_ = 54, N_st_ = 15, P = 0.04; G62: U = 35, N_so_ = 13, N_st_ = 15, P<0.001; G68: U = 783, N_so_ = 55, N_st_ = 20, P<0.001). However, in pair-wise comparisons within birds for different noise conditions, individual subjects differed in how much more oxygen was consumed during song than during inactivity (Table 3). For example, in all four background noise conditions, bird G68 had significantly higher 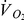 during song than during inactivity (Fig. 3c). In contrast, while mean values of O_2_ consumption during song were higher than during inactivity, for birds G16 and G62, these differences were only statistically significant in the intermediate noise condition (74 dB) (Fig. 3a & b).

**Table 3:** Mean song amplitudes, mean rates of oxygen consumption during song, and song – inactivity O_2_ consumption in three background noise conditions (no-noise, 54 dB(A); intermediate noise, 74 dB(A); and high noise, 80 dB(A)).

		Song amplitude (dB(A)/O_2_ consumption (ml/min/g)			Song - Inactivity O_2_ consumption (ml/min/g)		
		Background noise db(A)			Background noise db(A)		
Bird ID	Weight (g)	54	74	80	54	74	80
G16	14.02	79.9/0.212	97.8/0.236	106.1/0.266	0.005	0.031	0.013
G62	18.19	88.0/0.210	98.6/0.211	108.4/0.217	0.051	0.045	0.030
G68	18.59	80.8/0.272	100.7/0.200	108.5/0.276	0.086	0.030	0.029

**Figure 6:**
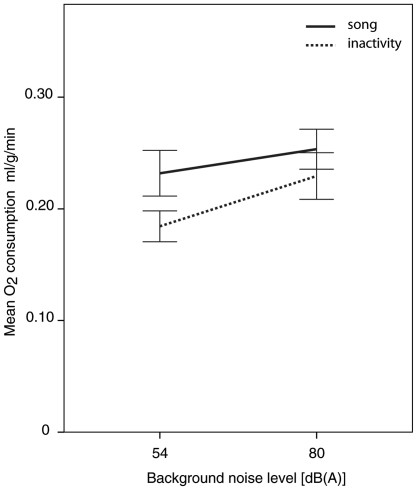
Mean 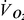 during song (solid line) and during inactive periods (dashed line) for all three birds at lowest and highest background noise conditions. Error bars ±1 s.e.m.

Since energy expenditure during periods of inactivity increased significantly between the no-noise and loud noise conditions, we compared the slopes of the change in 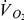 between song and inactivity at the lowest (54 dB(A)) and highest (80 dB(A)) noise conditions (Figure 6). The increase in 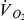 during active periods was not different from the increase in 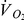 during song at the lowest and highest noise conditions (GLMM: N = 3; χ^2^(1) = −53.3; P = 0.22).

### Song vs. low-level activity

In the no-noise condition (54 dB) and the loud noise condition (80 dB) there was no significant difference in 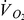 between song and low-level activity in all three birds. In addition, 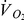 values during song and activity at intermediate sound levels (66–74 dB) were not significantly different in birds G16 and G68. Oxygen consumption during activity at intermediate noise conditions was statistically lower than during song in the same background conditions (Mann-Whitney U test; U = 13, P = 0.01). For bird G68, 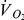 during activity followed the same pattern as it did for song in this bird, with rates of consumption decreasing during intermediate noise conditions relative to no-noise and loud-noise rates.

## Discussion

While the difficulty of recording song at different amplitudes from birds encumbered by wearing respirometry masks meant a limited sample size, our results do suggest that raising bird song amplitude, even by 20 dB, does not require consistent or significant increases in oxygen consumption. However, we did find that subsyringeal air sac pressure significantly increased with increasing song amplitude. Greater air sac pressure would be accomplished by an increase in respiratory muscle activity (Goller and Cooper, 2004), which would, in turn, result in additional metabolic energy requirements, it is likely that there is some metabolic cost to singing louder, but any such increases in metabolic energy consumption were not large enough to detect above the background metabolism. While we did find significantly higher rates of O_2_ consumption during the loudest songs than during the quietest songs in one bird, the increase in 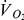 during inactive periods and low-level, non-vocal activity for the same background noise conditions were as great or greater than the increase during song. In addition, 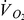 during low-level activity at each background noise level followed the same pattern, and did not differ statistically from O_2_ consumption during song. The comparison with energy requirements during inactivity, activity and song at low and high background noise levels suggests that any apparent increase in energy requirements for loud song can be explained by factors other than song amplitude per se.

The metabolic costs of bird songs and calls have been previously examined with mixed results. Previous studies attempting to measure the costs of vocal behaviour in birds vary greatly in their results, with estimates of the metabolic costs ranging from 0 to 9 times the basal metabolic rate, or the rate required for perching quietly (e.g., Chappell et al., 1995; Eberhardt, 1994; Gaunt et al., 1996; Horn et al., 1995; Oberweger and Goller, 2001). However, it is likely that these differences in energy estimates reflect extreme differences in methodology more than real differences in the oxygen consumption needed for vocalization. In particular, most studies have measured consumption of birds inside sealed respirometry chambers and the ratio of chamber volume to flow rate has often been too large to resolve brief changes in metabolic rate. To get around this limitation, Franz and Goller [23] developed a very small, lightweight respirometry mask for zebra finches, and using a very high flow rate were able to measure O_2_ consumption with the temporal resolution necessary to isolate the metabolic costs of individual songs from other activity immediately before and afterwards. Their study found that the O_2_ consumption during song is closely linked to the O_2_ consumption immediately before song began, but variation in pre-song O_2_ consumption was at least four times greater than the mean difference between song and pre-song rates. This suggests that estimations based on factorial increases in metabolic rate between inactivity and song, such as those reported in previous studies, are not reliable. We used a similar protocol as that used by Franz and Goller [23], but as we needed accurate measurements of vocal amplitude in addition to oxygen consumption, we designed a slightly larger mask (helmet) system that could include a tiny microphone, and that allowed freer movement of the beak and head of the bird. While the increased volume of our mask (10 ml vs. their 1 ml) meant a slight reduction in temporal resolution, we were still able to track changes in oxygen consumption at the song bout level.

In humans increasing the SPL of speech above “comfortable” levels has been shown to significantly increase oxygen consumption and metabolic energy consumption. When speaking at a “comfortable” sound pressure level in a quiet room, rates of oxygen consumption were not greater than those during quiet respiration, and ventilatory homeostasis was maintained [31]. In our study we found that rates of O_2_ consumption during song at low levels were not higher than those during “quiet respiration” in two of the three birds. However, unlike the human speakers, who continued to sit still while speaking, directed zebra finch songs are usually accompanied by courtship display including stereotyped body, head and beak movements [37], and the added energy required for these movements are likely to contribute to the metabolic cost of song. This assumption is supported by our observation that O_2_ consumption during low-level activities was not significantly different than consumption during song at ambient room noise levels.

Although speech in humans at comfortable speaking amplitudes is not energetically costly, when the human subjects were asked to repeat the same vocalizations at higher SPL (+10 dB above comfortable levels) energy expenditure significantly increased and homeostasis was disrupted [31]. We expected to find a similar pattern of increased O_2_ consumption in zebra finches singing at higher SPL, but O_2_ consumption was only significantly different between the lowest and highest amplitude conditions in one bird. However, even the apparent increase in O_2_ consumption for the loudest songs in this bird is probably due to other factors since consumption during inactive periods increased as well, so that the energy required for song in noise was not significantly greater than that required for quiet respiration in noise.

We observed that O_2_ consumption during periods of inactivity significantly increased in response to playback of noise in all three birds. This increase in metabolic consumption in response to noise playback suggests that exposure to noise itself may contribute as much, if not more, to the cost of singing in noisy environments as increasing vocal amplitude does. However, in contrast to the birds in our experiment, birds that live in areas where they are chronically exposed to high levels of anthropogenic noise may have habituated to the noise to the extent that they do not show the same elevation in metabolic rate as our experimental birds did. In addition, birds in areas with chronically high noise levels regularly sing louder than conspecifics in quieter habitats [38], and they might have learned to produce loud song more efficiently, just as trained human singers learn to improve vocal efficiency [33], [39].

Within each individual, louder renditions of the same songs required significant increases in subsyringeal air sac pressure than quieter ones. Previous studies have demonstrated a positive correlation between air sac pressure and relative amplitude of syllables within a song or the relative amplitude during a single syllable (see [40]). However, to our knowledge, our study is the first to experimentally show that the same utterance produced at higher amplitudes requires higher subsyringeal pressure. Subsyringeal air sac pressure can be increased either by increasing activity or contraction strength in the respiratory muscles. Goller and Suthers [41] demonstrated that electromyogram activity in both the external oblique and abdominal transverse muscles increased with increases in air sac pressure during birdsong. Another mechanism by which subsyringreal air sac pressure and sound amplitude can be increased is by reducing the aperture of the syrinx through which the air is expelled. This mechanism, however, would also necessarily change the frequency of the sound emitted because it requires an adjustment of the tension of the vibratory tissues in the syrinx. Since we did not find that louder zebra finch songs differed in fundamental frequency from quieter sounds, we conclude that the elevation in air sac pressure must be the result of increased respiratory muscle activity. It is nonetheless possible that the energy required for respiratory muscle activity during song represents only a small percentage of the total metabolic costs of singing, and therefore even significant increases in respiratory motor activity do not significantly impact the overall rates of oxygen consumption.

While our results suggest that the direct metabolic cost of singing significantly louder may not be very large per song bout, many birds, including zebra finches, may sing hundreds of songs per day, so even very small increases in energetic costs may translate to a larger cost over the course of a day or breeding season. In addition, there are a number of non-metabolic costs potentially associated with song amplitude. One way that birds are likely to regulate song amplitude is by adjusting subsyringeal air sac pressure [35]. Higher air pressure from the lungs, and increased rate of air flow through the syrinx, would increase vocal amplitude, but would also mean that the bird will exhaust its respiratory air more quickly. However, we did not find correlations between increasing song amplitude and duration or depth of minibreaths, or periods of post-song apnea. Another potential physiological cost could result from wear and tear on the vibratory tissues within the syrinx. In humans, vocal strain caused by sustained high amplitude vocalization can result in inflammation of the vocal folds, and temporary loss of voice or hoarseness, and can lead in more severe cases to vocal polyps [42], [43], [44], [45]. The vibratory tissues in the syrinx are different in composition than the mammalian vocal folds: the medial and lateral labia and tympaniform membranes are comprised mostly of elastic and collagen fibres [46], and may be less prone to inflammation from mechanical stress or trauma than the more heavily vascularized muscle tissue comprising the deep layer of mammalian vocal folds. Nonetheless, it is still unclear whether the vibratory tissues in the songbird syrinx might suffer adverse effects as a consequence of unusually loud song over sustained periods. Even without tissue damage, vocalizing at the limits of the individual's vocal range may result in degradation of vocal quality. For instance, in zebra finches and nightingales, the amplitude of different song elements is regulated to different degrees, so that low-amplitude elements are amplified much more strongly than high-amplitude ones when the singing bird increases its overall song level, and this results in less varied amplitude modulation patterns in loud songs [14], [15]. Moreover, an increase in song amplitude to levels close to the physical limits of song production may lead to an increase in vocal “roughness” resulting from nonlinear dynamics of the vibratory tissues in the syrinx. Increased occurrence of nonlinear vocal phenomena has been observed in several species of mammals when vocalizing at the extremes of their vocal dynamic range or in situations of high arousal (e.g. [47], [48], [49], [50]) and vocal hoarseness resulting from vocal fatigue has also been described in fallow deer (*Dama dama*) [51]. However, it is still unknown whether there are similar consequences for sustained high-intensity vocalizations in birds. In addition to these potential physiological costs, louder song will transmit further than quieter song, and thus could incur the cost of attracting unwanted attention from predators or rivals further afield [13], [52]. In addition to sound detection by unwanted receivers, social aggression is very likely to limit the performance of loud songs because high-amplitude songs elicit stronger aggression by rival males [53].

To summarize, even substantial increases in song amplitude (>20 dB) did not require significant increases in oxygen consumption compared with quieter song, but did require increases in air sac pressure. Although we could not detect an increase in metabolic cost for singing more loudly with our current measurement system, the increase in air sac pressure indicates that there is probably an additional metabolic cost over quieter songs but these costs appear to be very small, especially in comparison to the daily energy budget of a small songbird. In addition, these results also suggest that the generation of increased airflow by increased air sac pressure may account for only a small fraction of the energetic cost of singing. Other motor activity (e.g. that which controls the syrinx, upper vocal tract, or courtship displays), and neural processing must be significant other components that may not change much in different background noise conditions. Nonetheless, we suggest our results should encourage researchers to be cautious about making assumptions as to the costs of increasing vocal amplitude. While our data support previous observations that high levels of anthropogenic noise can induce physiological stress responses, and may even elevate metabolic rate, these effects appear to be independent of the costs associated with vocalizing at louder levels in birds. Further research is needed to identify other non-metabolic costs or consequences of increasing song amplitude and also the effects of chronic noise exposure.

## Materials and Methods

### Birds and Song

Adult male zebra finches (>1 year old) were used for these experiments, which were conducted during 2008 and 2010. Birds were from stock bred in a flight aviary at the University of Utah. In 2008 all birds were from North American breeding stock, but in 2010 we also used zebra finches that were first generation hybrids between American birds and imported zebra finches from the United Kingdom which are much larger in body size than American birds. These hybrid birds were used in the respirometery studies with the hope that their larger size would allow them to habituate more readily to wearing the respirometry helmets (See Table 1 for details on bird breeding stock and body mass). During the experiments, birds were held individually in 32×23×23 cm wire cages on a 13 h: 11 h L∶D cycle. We elicited directed song [37] from experimental birds by placing a caged female immediately in front of the subject. All experimental protocols were approved by the Institutional Animal Care and Use Committee of the University of Utah.

Zebra finch song consists of stereotyped sequences of individual syllable types (“motifs”), which are often repeated several times in quick succession to form a song “bout” (Figure 4B). Bouts are usually preceded by a series of short introductory notes. During song, zebra finches take short “minibreaths” between adjacent syllables to replenish air used during phonation [40]. These minibreaths can be seen in the air sac pressure recording (shaded blue in the first motif of Figure 4B), as the pressure goes negative during inspirations and positive during expiration and during phonation of most syllable types.

### Oxygen Consumption Measurements

**Figure 7:**
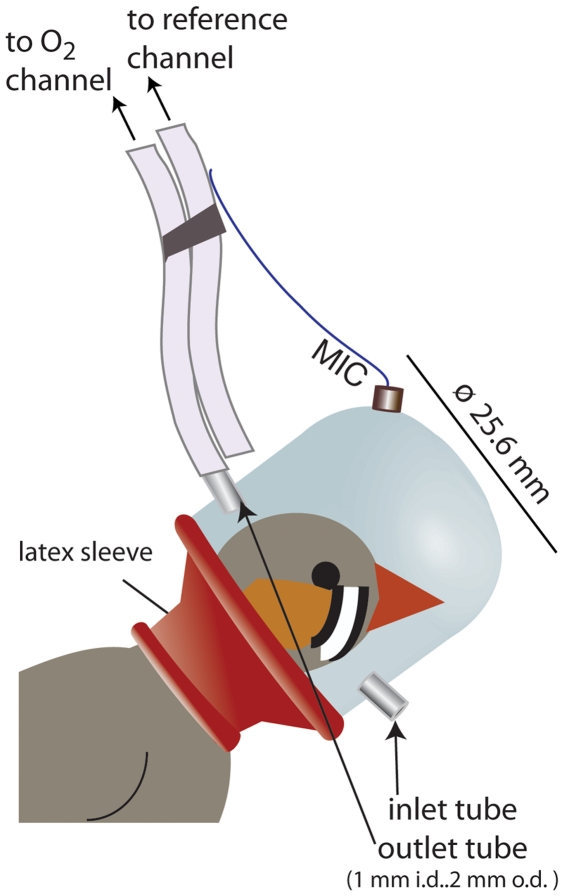
Schematic illustration of the respirometry helmet used to measure oxygen consumption (additional details in text).

In order to resolve the oxygen consumption required for individual songbouts (≥0.3 sec) from pre- and post-song activity, we needed a respirometry “chamber” with the smallest volume possible. We therefore designed small, lightweight respirometry masks, or more accurately, helmets that fit over the birds' heads (Figure 7). We trained birds to wear the helmets by first fitting them with a training helmet of the same proportions as the respirometry helmets, but which were not tethered to the oxygen analyser and amplifier, and had holes cut into the plastic shell to allow the bird to breathe. Small weights were added to the training helmets every day until the birds were comfortable wearing helmets of the same mass as the instrumented respirometry helmets and were singing while in these training helmets (5–10 days). They were then ready for the experiment.

Helmets were constructed of thin clear plastic domes (10.8 ml volume, 25.6 mm inner diameter, 25.6 mm height). A latex rubber sleeve (the neck of a balloon) was attached to the bottom of the helmet as a collar, which was stretched to fit over the head of the bird and fit snugly around the neck. In addition, a thin belt made of elastic and Velcro® was used to hold the helmet securely in place.

Experimental helmets were the same dimensions as the training helmets, but were instrumented with a subminiature performance microphone (2.56×2.56 mm, 0.08 g, FG-23329-C05, Knowles Electronics, Itasca, IL, USA). Two short (4 mm), blunt lengths of syringe needle (1.2 mm i.d., 1.7 mm o.d.) were inserted on opposite sides of the helmet as air inlet and outlet tubes. The helmets weighed 1.06 g before and 2.20 g after the addition of microphone, inlet and outlet tubes and latex rubber neck. The tubes connecting the instrumented helmet to the oxygen analyser were taped to the wire leads from the backpack, allowing some flex in the tubing so that the bird could move his head, but also supporting some of the weight of the helmet to be borne by pull of the counterbalanced arm to which the wire leads were attached. This arrangement, along with the period in the training helmets, meant that the birds were able to stand erect and move about the cage while wearing the instrumented helmet.

To the outlet tube, we attached a 35 cm length of flexible Silastic ® laboratory tubing (1 mm i.d., 2 mm o.d.) through which the air was drawn from the helmet into the oxygen sensor. A second length of Silastic tubing of the same length was taped to the first, but was not attached to the helmet, and drew air from outside the helmet to the reference channel of the oxygen analyzer (see below). The air from both channels was pulled through parallel 10 cm columns of desiccant (Indicating Drierite, W.A. Hammond, Xenia, OH, USA). Short lengths (10 cm) of Tygon® tubing led from there to the oxygen analyser. The inlet tube allowed room air to be drawn into the helmet as spent air was drawn out for analysis.

A flow control unit (R-2; Applied Electrochemistry, Pittsburgh, PA, USA) was used to pull air through the analyzer. Flow rate was kept at 350 ml/min. The percentage difference in oxygen content between the ‘helmet’ channel and the ‘reference’ channel was measured with an Applied Electrochemistry S-3A/2 Oxygen Analyzer (N 37M sensor). The sensor was calibrated with room air (20.95% oygen), and all recordings were done at room temperature (22–24°C, 10–17% humidity).

Once the bird was placed in a helmet that was connected to the analyser, he was given a 30 minute adjustment period, during which no females were in view, before recordings were started. We stimulated birds to increase the amplitude of their song by playing white noise at 4 different sound pressure levels, utilising the Lombard effect to make the subjects change their vocal amplitude (Cynx et al., 1998). Background noise playback files were looped, 10 min long, white noise generated using Adobe Audition 3.0 (Adobe Systems Inc., San Jose, CA, USA), using 44.1 kHz sampling rate and 16-bit resolution. These digital noise files were played through an amplifier (Bogen GA-6A series v.10.91) and monaural speaker (Bogen FG-15B) positioned above the cage. The sound pressure level of noise inside the sealed helmet at the position of the single perch in the cage was measured with a calibrated digital SPL meter (Checkmate CM-140, Galaxy Audio, Witchita, KS, USA). Sound pressure level of the noise playback were not always constant between birds owing to differences in cage and speaker placement, distance of speaker to perch level or room acoustics. However, because we were interested in within-bird differences rather than between-bird differences, our goal was simply to elicit a range of song amplitudes from each bird by exposing them to low, intermediate, and high background noise levels rather than to carefully standardize the SPL levels of noise exposure across all experimental designs and between years.

### Air Sac Pressure Measurements

The instrumentation of the birds to measure air sac pressure followed established procedures [54], [55]. Once the birds were in individual cages and regularly singing with the training helmet on, they were fitted with an elastic belt that ran around the thorax, just caudal to the wings. A leash was attached to a Velcro tab on the back of the bird, which was sewn to the elastic belt. The leash ran through the top of the cage to a counterbalanced tether arm, so that the bird was free to move around the cage while tethered.

Once singing resumed (1–3 days after males were tethered), birds were surgically implanted with a Silastic tube cannula (0.76 i.d., 1.65 o.d., 6 cm length) in one of the thoraco-cranial air sacs. Birds were deprived of food and water for 1 hour pre-surgery. They were then anesthetized with Isoflurane (Halocarbon Products Corporation, River Edge, NJ, USA) and a small hole was made in the abdominal wall into either the left or right cranial thoracic air sac. The flexible tip of the Silastic tube cannula was inserted into the hole and sutured to the caudal-most rib. The skin around the cannula insertion site was then sealed with tissue adhesive (VetBond, 3M, St. Paul, MN, USA) to prevent air leakage. The free end of the cannula was connected to a small piezoresistive pressure transducer (FHM-02PGR-02, Fujikura, Tokyo, Japan), which was attached to the Velcro tab on the bird's belt. The voltage output from each pressure transducer was calibrated before and after each experiment with a digital manometer (HHP-90, Omega Engineering, Stamford, CT, USA). In addition to recordings made from birds wearing respirometry helmets, we recorded song amplitude and calibrated air sac pressure from eight unhelmeted birds. The air sac cannulae can become clogged, or coated with fluids and connective tissue after a few days. We controlled for this degradation in signal by comparing the amplitude of the pressure traces during quiet respiration, and discarding data when this amplitude began to decrease, typically 2–5 days after implantation. Only five of the eight birds sang at multiple song amplitudes during the period when their air sac pressure recordings were usable, so only these birds were used in analyses of calibrated pressure. Of the eight unhelmeted birds from which we had calibrated pressure, six were suitable for analyses of minibreath size (Table 1).

### Recording of songs and data collection

Several different methods were employed to collect song amplitude data. Three birds were recorded singing at different amplitudes using the subminiature microphones inside the respirometry helmets. In addition, we recorded song at multiple amplitudes from the unhelmeted birds involved in the air sac pressure recordings by a directional microphone (AT835b, Audio-Technica U.S., Inc., Stow, OH, USA) held at a fixed location ∼35 cm above the perch.

We wanted to rule out the possibility that helmet-wearing or air sac cannulation might inhibit the Lombard effect in zebra finches. We therefore recorded song at different background noise conditions using the same subminiature microphones and recording equipment used with the helmets, but in a setting where they birds had not been surgically implanted with cannulae, were not tethered and were not wearing helmets. This was achieved by recording the birds in small translucent chambers, which had only a small (1.5 cm square) transparent window through which the bird could see a nearby female. The chambers had been designed to be used as respirometry chambers in a previous unsuccessful attempt to measure oxygen consumption during song. The birds had to stand on a perch and stick their head and breast into a cylindrical tube (50 mm diameter) in order to see and sing to a female bird through the window. We fixed a microphone identical to those used in the helmet recordings to the ceiling of the tube in a position that was directly above the head of the birds when they were perched and singing through the window. The small window and confining size of the tube kept the singing male in a relatively fixed position relative to the microphone and allowed the microphone to be placed less than 2 cm from the bird's head. This allowed both accurate measurements of song amplitude, and measurements that simulated in many ways the recording conditions inside the helmets.

In helmeted birds and birds in chambers, the output from the microphone was amplified (Model 410, Brownlee Precision Co., Palo Alto, CA, USA). The amplified microphone output and voltage signals from the pressure transducer and oxygen analyser were digitally recorded simultaneously into three channels of Avisoft-RECORDER (v. 3.4, Avisoft Bioacoustics, Berlin, Germany) with 1-bit resolution and a 44.1 kHz sampling rate.

### Song Amplitude Analysis

To measure the sound level of the songs we calibrated Avisoft with recordings of unmodulated 2 kHz tones or 10 second periods of white noise of known sound pressure levels, measured inside the sealed helmet with the SPL meter. Calibration sounds were recorded with the same recording equipment and settings at the beginning and end of each recording session for each background noise condition. We measured the mean peak amplitude (the average of the 3 loudest elements) of each song motif (root mean square (rms) values with 125 ms averaging time), from three birds with helmets (Bird G16, 57 songbouts, 350 motifs; G62, 13 songbouts, 24 motifs; G68, 55 songbouts, 122 motifs).

The sound level of the background noise was subtracted from the total amplitude measurements using the logarithmic computation procedure given by Brumm et al. [56] in order to calculate the sound pressure level of the song (L_signal_ ):
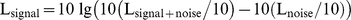
where L_signal+noise_ is the sound pressure level of the signal and the noise, and L_noise_ is the sound pressure level of the noise alone.

Since the helmet enclosed the entire head of the bird, including its ears, we wanted to verify that the birds would still increase song amplitude in response to an increase in environmental noise. The helmet might interfere with the Lombard effect by disrupting the normal auditory feedback to the bird of his own song amplitude, or by inhibiting or distorting the bird's perception of the external acoustic environment.

### Oxygen Analysis

We used custom-written software developed in MatLab (2007a, The MathWorks, Natick, MA, USA) to calculate mean oxygen consumption during selected time intervals. The mean rate of oxygen consumption was calculated from the output of the oxygen analyzer using the equation of Withers [57] for a closed mask system,
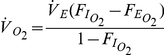
Where 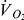 is the rate of oxygen consumption, 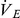 is the rate of airflow being pulled through the mask, 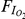 is the fractional concentration of oxygen entering the mask and 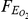 is the fractional concentration of oxygen leaving the mask.

We recorded oxygen consumption during song bouts (“song”), during periods when the bird was sitting quietly on the perch or cage floor without interruption for at least 20 minutes with no female in view (“inactivity”), and during “activity” (low-level activity such as hopping around cage, scratching, fluffing feathers, but without vocalizations). Females were not in view during “activity” measurements, but were sometimes in a neighbouring cage that could be heard by the test bird. Because birds were rarely sitting quietly just before singing and because visual contact with a female was likely to elevate arousal and thus might affect energy expenditure, we used the mean oxygen consumption measured during inactive periods as a baseline for metabolic energy consumption at each noise playback condition. Although during inactive periods the bird appeared to be doing nothing other than respiring quietly, the lack of observable activity in the bird is not meant to imply that these measurements are indicative of the bird's true resting metabolic rate (RMR), which we did not measure. Zebra finch song consists of song bouts made of one to many repetitions of song motifs, which are composed of a fixed sequence of sound elements or syllables. Syllables were considered part of the same motif if there were less than 50 ms silence between them, and motifs were considered part of the same song bout if there were less than 200 ms of silence between them. Since the duration of song bouts within individuals varied considerably between songs, we standardized the oxygen consumption at different amplitudes by using the per-motif consumption. In all but one case (bird G68, 74 dB background noise), we were able to record song, activity, and inactivity during more than one recording session to control for potential order effects of the different background noise treatments.

We were interested in how changes in song amplitude would affect the rate of O_2_ consumption within each individual, and therefore the absolute song amplitudes produced by each bird were less important than a significant increase in song amplitude for each increase in background noise condition. Although the song amplitudes produced by birds wearing helmets potentially differed from song amplitudes that they might have produced under similar noise conditions outside the helmet, for convenience we refer to songs produced in the “no noise” (54 dB) playback condition as “normal” amplitude songs. Songs produced when noise playback was between 66 and 74 dB we refer to as “intermediate” amplitude songs and songs produced during 80 dB noise as “loud” songs.

### Statistical Analyses

Statistical tests were performed with SPSS (IBM SPSS Statistics rel. 18.0.0, Chicago, IL, USA) or with R 2.10.1 (R Development Core Team, 2009). The function lmer (R package lme4) was used to fit generalized linear mixed-effects models (GLMM) with individual subject as a random factor to account for repeated sampling of the same individuals (at low and high background noise conditions), and song amplitude, song duration or post-song apnea duration as the dependent variable, and helmet wearing, background noise or song duration included as fixed factors, respectively. We used Wald χ^2^ tests to investigate whether there was a significant interaction between the fixed factor and the dependent variable. Likelihood ratio tests were used to compare the deviance of the model containing the main effects, background noise level (an ordinal factor with 2 levels, 54 and 80 dB) and behaviour (a factor with two levels: inactivity and song) and the second order interaction between these effects with that of the model comprising just the main effects. If the interaction was not significant, we removed it from the model, leaving us with the model only containing the two main effects. We tested the significance of main effects by removing factors one at a time and comparing the model with only one main effect to the model with both main effects, also using the likelihood ratio test as described above.

Differences in 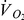 within birds at different background noise conditions were investigated using Kruskal-Wallis H tests in SPSS, with pair-wise comparisons done using Mann-Whitney U tests. Differences in air sac pressure required to sing the same syllable at different vocal amplitudes within a bird were investigated using one-way repeated measures ANOVA, with pair-wise comparisons made using the Holm-Sidak Method in SPSS.
